# Correction: BMP2 expression in oral squamous cell carcinoma and its effects on SCC9 cell biological behavior

**DOI:** 10.1038/s41598-025-00862-1

**Published:** 2025-05-27

**Authors:** Fuao Xing, Yimin Liu, Faming Tian, Xiaoli Hou, Qiangqiang Lian, Yunpeng Hu, Lei Xing, Jing Yuan Gao, Xinhao Fan

**Affiliations:** 1 North China University of Science and Technology, Tangshan, 063000 HeBei China; 2 Affiliated Hospital of North China University of Science and Technology, Tangshan, 063000 HeBei China; 3 Affiliated Kailuan General Hospital of North China University of Science and Technology, 57 Xinhua East Road, Tangshan, 063000 HeBei China; 4 Second Hospital of Tangshan, Tangshan, 063000 HeBei China; 5 School of Dental Sciences, Universiti Sains Malaysia, 16150 Kubang Kerian, Kelantan Malaysia

Correction to: *Scientific Reports* 10.1038/s41598-025-96274-2, published online 04 April 2025

The original version of this Article contained errors where Fig. 5 was a duplication of Fig. 4. As a result, the designated Fig. 5 was omitted. Furthermore, Fig. 4 contained errors where a previous version of this figure was published. The original Figs. 4 and 5 and accompanying legends appear below.

**Fig. 4:**
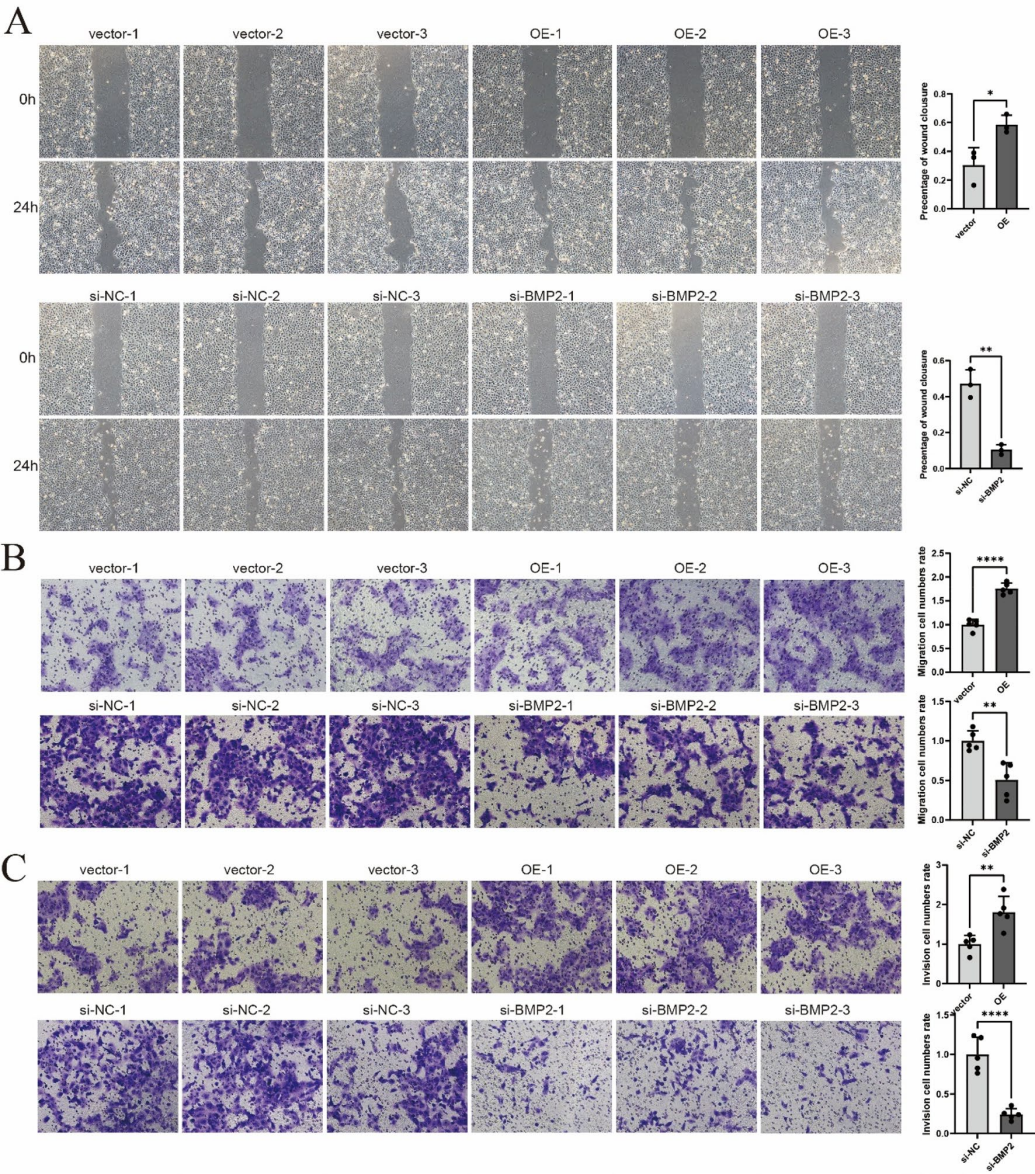
BMP2 knockdown suppresses SCC9 cell migration and invasion, whereas its overexpression enhances these oncogenic properties. **A** Post-transfection wound healing assay assessing OSCC cell migration capacity. **B** Quantitative assessment of migratory potential through Transwell migration assays following transfection. **C** Matrigel-coated Transwell invasion assays with quantitative analysis of invasive capability post-transfection. *p < 0.05, **p < 0.01, ****p < 0.0001

**Fig. 5:**
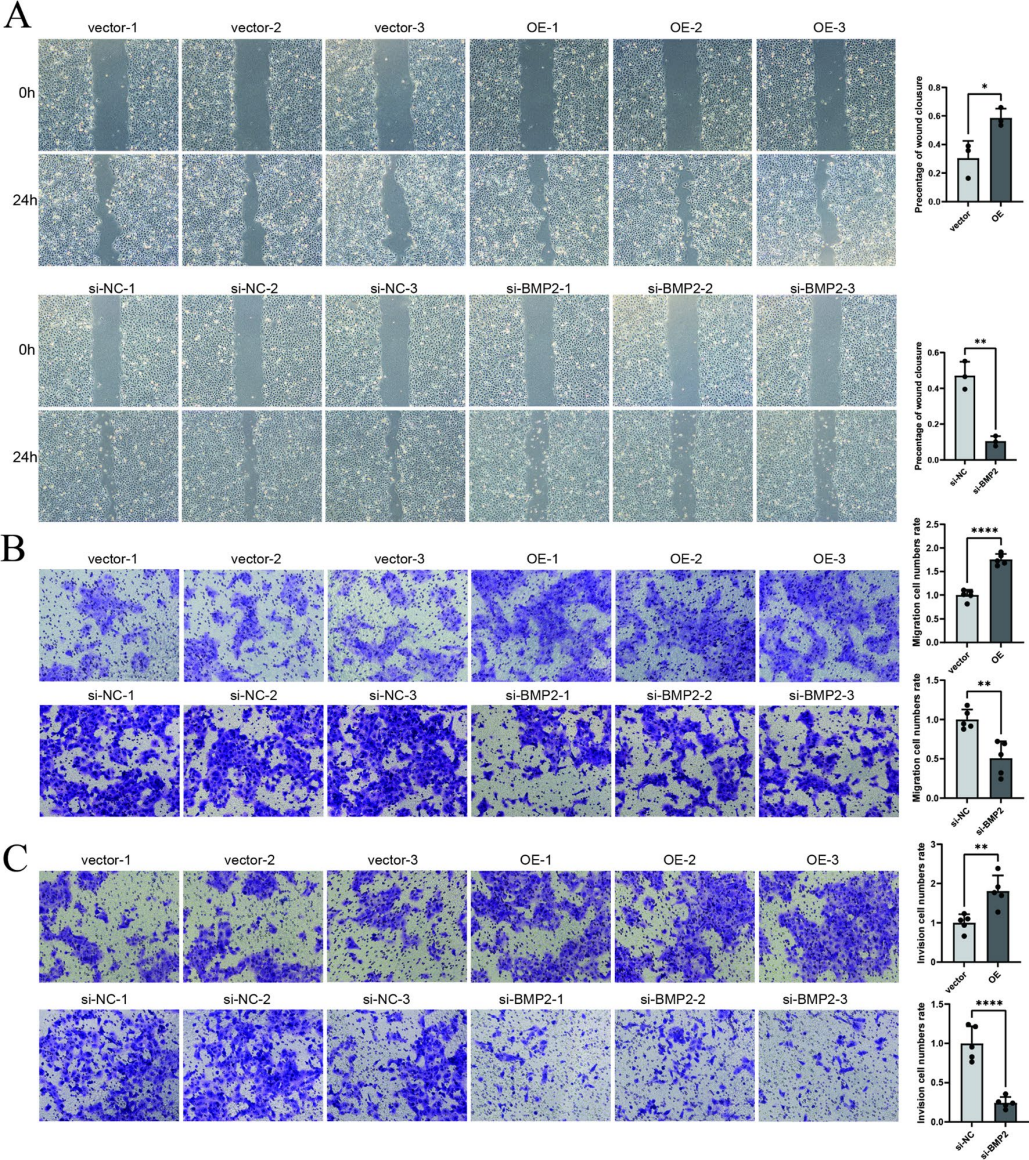
Flow cytometric quantification of Annexin V-FITC/PI dual staining demonstrated augmented apoptotic cell fractions in BMP2-knockdown populations (si-BMP2 vs si-NC controls), with conversely attenuated apoptosis observed in BMP2-overexpressing cohorts (OE vs vector controls). Representative density plots illustrate distinct quadrant distributions corresponding to viable (Annexin V^−^/PI^−^), early apoptotic (Annexin V⁺/PI^−^), and late apoptotic (Annexin V⁺/PI⁺) cellular states. *p < 0.05

The original Article has been corrected.

